# Antisense oligonucleotide-induced alternative splicing of the *APOB *mRNA generates a novel isoform of APOB

**DOI:** 10.1186/1471-2199-8-3

**Published:** 2007-01-17

**Authors:** Bernard Khoo, Xavier Roca, Shern L Chew, Adrian R Krainer

**Affiliations:** 1 Endocrinology, William Harvey Research Institute, Queen Mary University of London, Charterhouse Square, London EC1M 6BQ, UK; 2 Cold Spring Harbor Laboratory, Cold Spring Harbor, New York 11724, USA

## Abstract

**Background:**

Apolipoprotein B (*APOB*) is an integral part of the LDL, VLDL, IDL, Lp(a) and chylomicron lipoprotein particles. The *APOB *pre-mRNA consists of 29 constitutively-spliced exons. APOB exists as two natural isoforms: the full-length APOB100 isoform, assembled into LDL, VLDL, IDL and Lp(a) and secreted by the liver in humans; and the C-terminally truncated APOB48, assembled into chylomicrons and secreted by the intestine in humans. Down-regulation of APOB100 is a potential therapy to lower circulating LDL and cholesterol levels.

**Results:**

We investigated the ability of 2'O-methyl RNA antisense oligonucleotides (ASOs) to induce the skipping of exon 27 in endogenous *APOB *mRNA in HepG2 cells. These ASOs are directed towards the 5' and 3' splice-sites of exon 27, the branch-point sequence (BPS) of intron 26–27 and several predicted exonic splicing enhancers within exon 27. ASOs targeting either the 5' or 3' splice-site, in combination with the BPS, are the most effective. The splicing of other alternatively spliced genes are not influenced by these ASOs, suggesting that the effects seen are not due to non-specific changes in alternative splicing. The skip 27 mRNA is translated into a truncated isoform, APOB87_SKIP27_.

**Conclusion:**

The induction of APOB87_SKIP27 _expression *in vivo *should lead to decreased LDL and cholesterol levels, by analogy to patients with hypobetalipoproteinemia. As intestinal *APOB *mRNA editing and APOB48 expression rely on sequences within exon 26, exon 27 skipping should not affect APOB48 expression unlike other methods of down-regulating APOB100 expression which also down-regulate APOB48.

## Background

Apolipoprotein B (*APOB *– OMIM 107730) is the principal structural apolipoprotein in the LDL, VLDL, IDL, Lp(a) and chylomicron lipoprotein particles [1]. The *APOB *pre-mRNA comprises 29 exons, all constitutively spliced into the mature mRNA. There exist two natural protein isoforms, the full-length APOB100 and the C-terminal truncation APOB48. APOB100 is synthesized in the liver, and this isoform is required for the assembly of VLDL, IDL, LDL, and Lp(a). Along with APOE, APOB100 is the ligand for the LDL receptor. Excess levels of the APOB100-containing particles LDL and Lp(a) have been implicated in atherogenesis [2]. APOB48 is generated by tissue-specific RNA editing of a CAA codon to a premature UAA termination codon in the intestine, and is identical to the N-terminal 48% of APOB100. Editing is performed by a protein complex known as the editosome, which consists of the catalytic subunit APOBEC-1 and accessory factors. The RNA editing site and mooring sequence necessary for editosome binding and function are within exon 26. The APOB48 isoform is required for chylomicron assembly and intestinal fat transport. As the APOB48 isoform lacks the C-terminal domain necessary for LDL receptor binding, chylomicrons are unable to bind via this receptor, and are cleared by interaction of APOE with the chylomicron remnant receptor [3].

Because of its central role in atherosclerosis, *APOB *has become a major therapeutic target. Down-regulation of APOB100 expression would be expected to decrease VLDL, IDL, LDL and Lp(a) levels, and therefore prevent the development of atherosclerosis. Over-expression of the rabbit APOBEC-1 enzyme in transgenic mouse livers has been shown to cause increased RNA editing, isoform switching from APOB100 to APOB48, and lowered LDL levels [4]. Other approaches have concentrated on the down-regulation of *APOB *mRNA levels, for example by chimeric antisense-oligonucleotide mediated RNase H degradation [5], and by RNA interference [6,7].

We hypothesized that modifying *APOB *splicing would cause the expression of an alternative isoform of APOB. We selected exon 27 as our target for three reasons. Firstly, translation of the *APOB *mRNA lacking exon 27 (skip 27 mRNA) would generate a C-terminally truncated isoform of APOB, APOB87_SKIP27_, which is similar to the C-terminal truncations seen in some patients with hypobetalipoproteinemia. Heterozygotes for these mutant alleles of *APOB *have lower cholesterol and LDL levels [8]. Secondly, the 5' splice-site of exon 27, when scored for its similarity to the splice-site consensus using the Shapiro and Senapathy position weight matrix, was the weakest of all the 5' splice-sites of the internal exons of *APOB *at 60.73 [9]. We reasoned that as constitutive 5' splice-sites in general score better than alternatively spliced 5' splice-sites [10], *APOB *exon 27 would be the most amenable to alternative splicing. Thirdly, as the sequences necessary for RNA editing are present in exon 26, the skip 27 mRNA should be edited as usual in the intestine, and APOB48 expression should be unaffected.

We demonstrate in this paper that the induction of exon 27 skipping causes the expression and secretion of a truncated isoform of APOB which is potentially of therapeutic utility in lowering LDL and cholesterol levels *in vivo*.

## Results

### ASO targeted to the splice-sites of APOB exon 27 induce exon skipping

**Figure 1:**
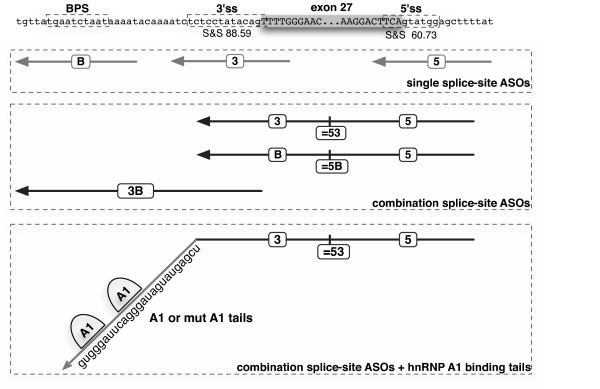
**Design of skip 27 ASOs**. The sequences of the intronic regions flanking exon 27 and parts of exon 27 are shown at the top – intronic sequences are in small letters, exonic sequences are in caps, exon 27 is enclosed in the grey-shaded box. The splice-sites (BPS = branch-point sequence; 3'ss = 3' splice-site; 5'ss = 5' splice-site) are outlined with dotted lines. The Shapiro and Senapathy (S&S) scores below each splice-site reflect the degree of similarity of these sequences to the splice-site consensus [9]. The ASO design is indicated below. Arrows run in the 5' to 3' direction. Three classes of ASO tested are distinguished within each box: single splice site ASOs that target individual splice sites; combination splice-site ASOs that target two splice-sites simultaneously; combination splice-site ASOs that target two splice-sites and are linked to an hnRNP A1 binding tail (A1 – the sequence of this tail is presented) or a mutated tail that is unable to bind hnRNP A1 (mut A1).

In order to modify exon 27 splicing, we used splice-site ASOs, which have previously been used to modify splicing in the dystrophin gene [11-13] and to correct the aberrant alternative splicing in the ß-thalassemia mutation IVS2-654 [14]. 2'O-methyl RNA ASOs were targeted to the 5' and 3' splice-sites of exon 27, as well as the branch-point sequence (BPS) just adjacent to the 3' splice-site of intron 26–27, to block their recognition by the spliceosome. The design of the ASOs is shown in Fig. 1. These ASOs were transfected into HepG2 cells, and total RNA was extracted and analyzed by RT-PCR with primers complementary to regions within exons 26 and 28.

**Figure 2:**
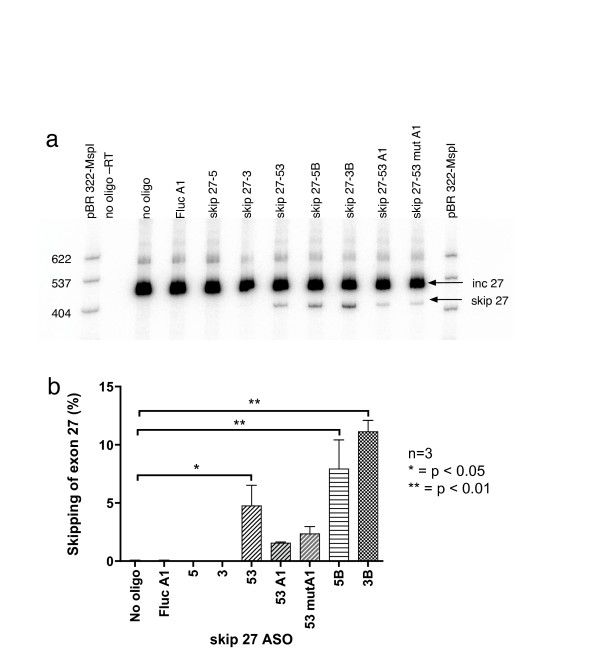
**Alternative splicing of exon 27 induced by *APOB *exon 27 splice-site ASOs**. (a) HepG2 cells were transfected with the indicated ASOs at 250 nM for 48 hours. RT-PCR was carried out on the total RNA extracted from these cells with oligonucleotides annealing to the adjacent exons 26 and 28. The -RT control PCR was performed without reverse transcriptase. The positions of the bands corresponding to the *APOB *mRNA species with exon 27 (inc 27) and without exon 27 (skip 27) are indicated on the right side. Fragment lengths in bp of pBR322-MspI markers are indicated on the left side. The band corresponding to skip 27 mRNA was cloned and sequenced to confirm that exon 26 is spliced to exon 28. (b) The graph shows the quantitative data from three independent replications of the experiment (error bars show the S.E.M.). Overall one-way repeated measures ANOVA p-value was < 0.0001, indicating statistically significant differences between groups. Dunnett's multiple comparison test was used to compare the no-oligo control versus ASO-transfected cells: statistically significant differences were found with skip 27–53, skip 27-5B, and skip 27-3B.

As shown in Fig. 2, mock-transfected cells (no oligo) showed no skipping of exon 27, consistent with this being a constitutively-spliced exon. Cells transfected with an unrelated ASO directed against the firefly luciferase gene (Fluc A1) did not show any skipping of exon 27. Surprisingly, the ASOs directed either to the 5' or to the 3' splice-site (skip 27-5 and skip 27-3 respectively) did not have any effect on splicing. However, when both 5' and 3' splice-sites were targeted by the same ASO (skip 27–53), a shorter RT-PCR product was obtained. This effect was stronger if either skip 27-5 or skip 27-3 was linked to a sequence targeting the BPS (skip 27-5B and skip 27-3B respectively). Cloning and sequencing confirmed that this shorter RT-PCR product consists of exon 26 sequence spliced to exon 28, and therefore corresponds to the skip 27 mRNA. An ASO targeting the BPS alone (skip 27-B) was not effective in inducing skipping (data not shown). Moreover, a mixture of either skip 27-5 or skip 27-3 with skip 27-B did not induce skipping, indicating that these ASOs do not act synergistically (data not shown).

We also tested ASOs tailed with an RNA sequence designed to bind the inhibitory splicing factor hnRNP A1, a strategy that was previously shown to increase the efficiency of alternative splicing in the *Bcl-x *pre-mRNA *in vivo *[15,16]. The Fluc A1 ASO, which bears the A1 tail, does not have any effect on exon 27 skipping, as noted above. Linking the skip 27–53 ASO with the A1 tail (skip 27–53 A1) appeared to decrease the skipping of exon 27 compared to skip 27–53 itself (Fig. 2). Mutation of the A1 tail such that it does not bind hnRNP A1 (skip 27–53 mut A1) restored its effects to some extent, suggesting that hnRNP A1 itself is interfering with the ability of the skip 27–53 ASO to induce skipping.

**Figure 3:**
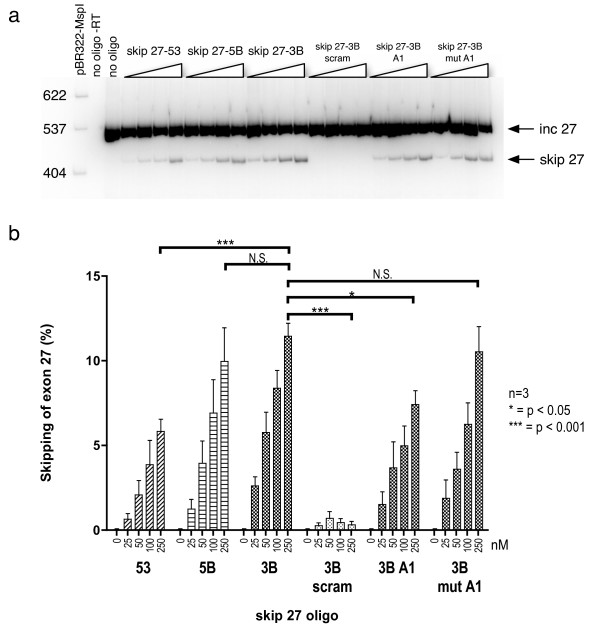
**Dose titration of selected *APOB *skip 27 ASOs**. (a) HepG2 cells were transfected with the indicated ASOs at increasing concentrations from 25 to 250 nM for 48 hours. RT-PCR was carried out on the total RNA extracted from these cells with oligonucleotides annealing to the adjacent exons 26 and 28. The -RT control PCR was performed without reverse transcriptase. The positions of the bands corresponding to the *APOB *mRNA species with exon 27 (inc 27) and without exon 27 (skip 27) are indicated on the right side. Fragment lengths in bp of pBR322-MspI markers are indicated on the left side. (b) The graph shows the quantitative data from three independent replications of the experiment (error bars show the S.E.M.). The data was analyzed using a repeated measures two-way ANOVA, using Bonferroni's test to compare exon 27 skipping between skip 27-3B and the other ASOs at 250 nM: statistically significant differences are seen with skip 27–53, skip 27-3B scram and skip 27-3B A1. No statistically significant difference is seen with skip 27-5B and skip 27-3B mutA1.

Fig. 3 shows that at concentrations ranging between 0–250 nM, the skip 27-3B ASO was consistently the most effective at inducing exon 27 skipping. A scramble mutation of skip 27-3B, skip 27-3B scram, had no effect even at the highest concentration, indicating that the alternative splicing effect of the skip 27-3B ASO is sequence-specific. Fluorescence microscopy of cells transfected with 5' fluorescein-labelled skip 27-3B and skip 27-3B scram ASOs showed that both ASOs were transfected with equal efficiency into ~80% cells, indicating that differences in transfection efficiency were not responsible for the different effects seen (data not shown). As before, tailing skip 27-3B with the A1 tail significantly reduced the efficiency of the ASO, as noted before with the skip 27–53 ASO. Mutation of the A1 tail (skip 27-3B mut A1) restored the efficiency of skipping to levels not significantly different from skip 27-3B, again suggesting that hnRNP A1 specifically interferes with the ability of the skip 27-3B ASO to induce skipping.

We conclude, therefore, that the 5' splice-site, the 3' splice-site, and the BPS are crucial for the constitutive inclusion of exon 27. ASOs targeting single splice-sites are not effective in inducing exon 27 skipping. Instead, combination ASOs that target two splice-sites are effective at inducing exon 27 skipping, and an ASO targeting both the 3' splice-site and the BPS (skip 27-3B) appears to be the most effective in doing so. As mixing individual single splice-site ASOs, for example combining skip 27-3 with skip 27-B, was not effective in inducing exon 27 skipping, it is unlikely that these ASOs work by simple occupancy of splice-sites and competition with splicing factors. Instead, we speculate that these ASOs might operate by inducing an alternative secondary structure in the *APOB *mRNA. An mFold analysis of the predicted conformation of exon 27 and its flanking intronic sequences [17] shows that the targeted 3' splice-site and BPS reside within relatively open structures [see Additional file 1]. As the secondary structure of RNA is known to influence splicing in some instances [18], it is possible that binding of the skip 27-3B ASO closes these open structures and discourages splice-site recognition by the spliceosome. Consistent with this idea, hnRNP A1 is known to unwind nucleic acid secondary structure [19], and this property may explain its paradoxical ability to restore exon 27 inclusion, perhaps by interfering with ASO hybridization to the *APOB *pre-mRNA (Figs. 2 and 3).

### ASOs targeted to putative exonic splicing enhancer motifs within exon 27 weakly induce its skipping

**Figure 4:**
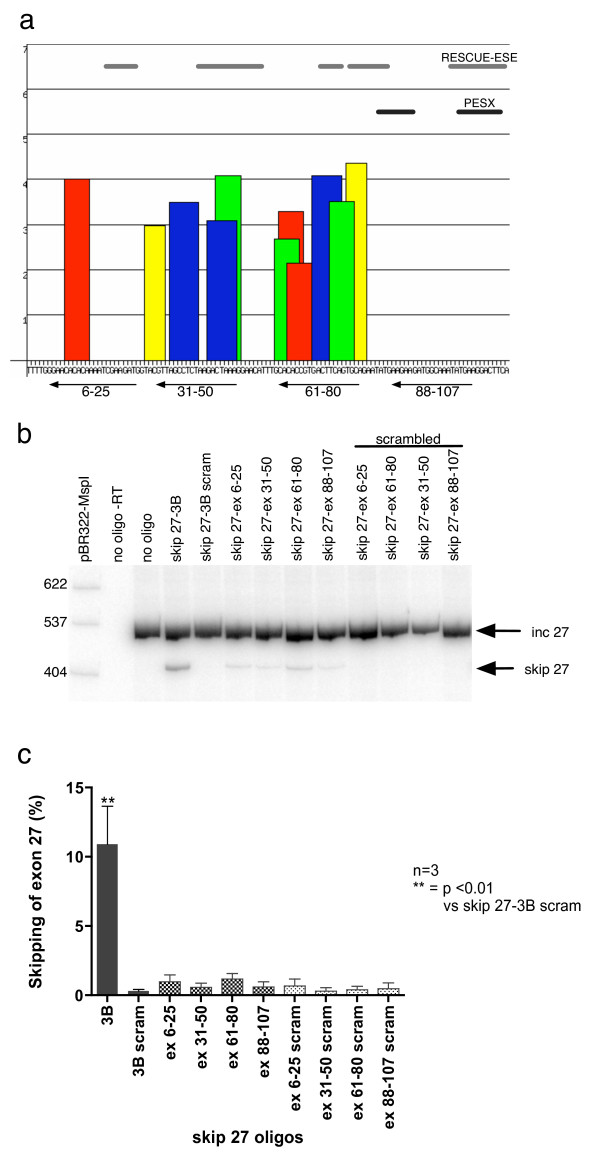
**Effect of ASOs targeting *APOB *exon 27 sequences**. (a) ESEfinder output for *APOB *exon 27, using version 2.0 matrices and thresholds [21]. Y-axis shows ESEfinder above-threshold scores for identified matches; X-axis corresponds to the exon 27 sequence. Red = SF2/ASF, blue = SC35, green = SRp40, yellow = SRp55. The light grey horizontal lines indicate the matches that were found by RESCUE-ESE [22]. The dark grey lines indicate the ESE candidates identified by PESXs server [23, 24]. ASOs were designed to bind to locations corresponding to ESE clusters, and these locations are represented below the X-axis by the arrows, which run from the 5' to 3' direction. (b) RT-PCR assay for exon 27 skipping with RNA from cells transfected with ASOs at 250 nM. Exonic ASOs 6–25, 31–50, 61–80, 88–107 weakly promote exon 27 skipping, whereas scrambled versions of these ASOs had no effect on exon 27 skipping. (c) Quantitative data from three independent experiments. Error bars show S.E.M. Overall repeated measures one-way ANOVA showed p < 0.0001, indicating significant differences between compared groups. Dunnett's multiple comparison test showed a significant difference between skip 27-3B and 3B scram (p < 0.01), but no statistically significant differences were seen between skip 27-3B scram and other skip 27 ASOs.

Exonic splicing enhancers (ESEs) are sequences within exons that activate inclusion of the exon that harbors them, typically by binding SR proteins [20]. We reasoned that ESEs within exon 27 might contribute to the constitutive inclusion of this exon, and that exon 27 skipping might be induced by interfering with SR protein binding to these elements. We turned to computational tools in order to identify potential ESEs: ESEfinder [21], RESCUE-ESE [22], and PESXs server [23,24]. Based on these predictions, four ASOs targeting nucleotides 6–25, 31–50, 61–80 and 88–107 were tested (Fig. 4a).

Fig. 4b shows that these ASOs induced skipping of exon 27, but much less efficiently than the splice-site ASOs described above. Furthermore, in order to test the idea that the exonic ASOs might synergize with each other, we transfected HepG2 cells with mixtures of the exonic ASOs. However, combinations of exonic ASO pairs and mixtures of all four exonic ASOs, although demonstrating some additive effects, were inefficient at inducing exon 27 skipping: the maximum exon 27 skipping demonstrated with mixtures of all four exonic ASOs was 1–2% (data not shown). We conclude, therefore, that the identified ESEs play a relatively small role in the inclusion of exon 27 during splicing.

### ASOs targeting the splice-sites of APOB exon 27 do not generally affect the splicing of other alternatively-spliced genes

**Figure 5:**
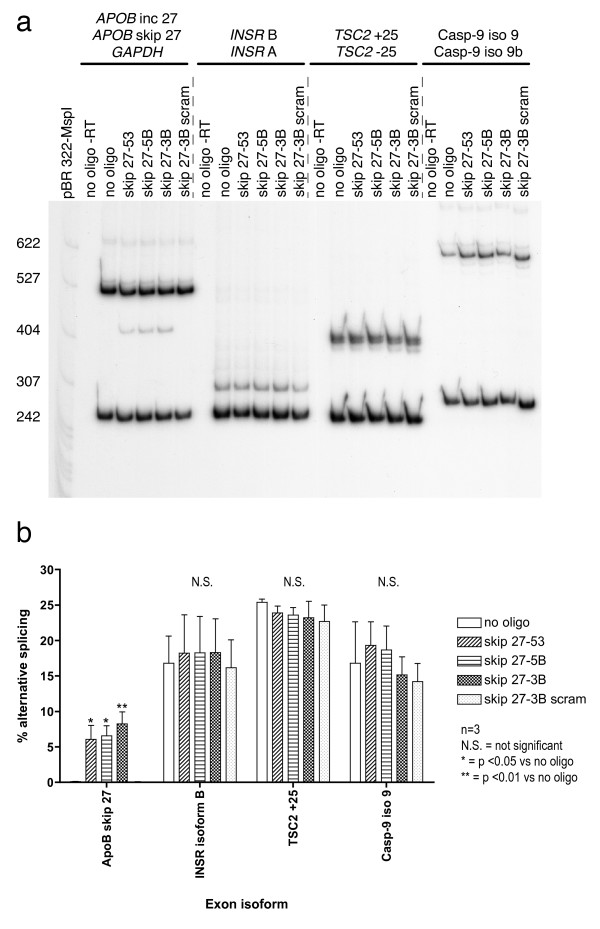
***APOB *skip 27 ASOs have no effect on the alternative splicing of other exons**. (a) The indicated ASOs were transfected at 250 nM into HepG2 cells, and total RNA from these was analyzed by RT-PCR for inclusion/skipping of *APOB *exon 27; *GAPDH*, as loading control; inclusion/skipping of insulin receptor (*INSR*) exon 11; inclusion/skipping of *TSC2 *exon 25; and inclusion/skipping of Caspase-9 exons 3–6. The identity of each band is listed below each set of 6 lanes from top band to bottom band: *APOB *inc 27 = exon 27 included; *APOB *skip 27 = exon 27 skipped, GAPDH = amplified region of GAPDH mRNA 7–261 nt; *INSR *B = exon 11 included, *INSR *A = exon 11 skipped; *TSC2 *+25 = exon 25 included, *TSC2 *-25 = exon 25 skipped; Caspase-9 isoform 9 = exons 3–6 included, Caspase-9 isoform 9b = exons 3–6 skipped. Transfection of ASOs does not affect the alternative splicing of *INSR*, *TSC2 *and Caspase-9. (b) Quantitative data for alternative splicing of each gene from three independent replicates. Error bars represent S.E.M. One-way repeated measures ANOVA of the *APOB *skip 27 dataset showed p = 0.0007, representing significant statistical differences between groups: Dunnett's multiple comparison test showed statistically significant differences between the no-oligo control and skip 27–53, 5B and 3B, as indicated. One-way ANOVA p-values for the *INSR*, *TSC2 *and Caspase-9 datasets were 0.3716, 0.5887, and 0.1674 respectively, indicating no statistically significant differences between groups.

Because splice-sites conform to a degenerate consensus, there exists a possibility that the *APOB *skip 27 splice-site ASOs might potentially bind to other pre-mRNA splice-sites. We searched for matches to the exon 27 splice-sites in a comprehensive database of splice-sites, SpliceRack [25], but did not identify any extra matches in the human database, indicating that this possibility is unlikely. In order to directly test the hypothesis that the splice-site ASOs might be non-specifically affecting alternative splicing, for example by binding splicing factors or by hybridizing to other pre-mRNAs, we tried to detect changes in the well described alternative splicing of other exons such as *TSC2 *exon 25, Caspase-9 exons 3,4,5 and 6, and insulin receptor exon 11 [26-29]. As shown in Fig. 5 there was no significant effect of skip 27 -53, -5B and -3B ASO transfection on the pattern of insulin receptor, *TSC2*, or Caspase-9 alternative splicing, suggesting that the effects of our ASOs on *APOB *exon 27 are not explained by a generalized and non-specific alteration of alternative splicing.

### The skip 27 mRNA is translated into a secreted polypeptide

The skip 27 mRNA encodes a truncated protein, APOB87_SKIP27_, which contains the N-terminal 3929 amino acid residues of APOB100 along with a divergent 37 amino acid peptide at the C-terminus, ending at a premature termination codon within the exon 28 sequence. Those mRNAs that harbor a premature termination codon (PTC) more than 55–60 nt upstream of the last exon-exon junction are subject to surveillance and down-regulation, in a process known as nonsense-mediated decay (NMD - [30]). The skip 27 mRNA's PTC is 71 nt upstream of the exon 28–29 junction, and is a candidate for down-regulation by NMD. We treated skip 27-3B ASO-transfected cells with cycloheximide, which is known to inhibit NMD in HepG2 cells [31,32]. Under these conditions we did not see up-regulation of the skip 27 mRNA, indicating that skip 27 mRNA appears not to be subject to down-regulation by NMD [see Additional file 2]. Although this is somewhat surprising, some potential substrates for NMD appear to escape NMD [33,34] and it is possible that the skip 27 mRNA contains a stabilizer element, similar in function to that found in the *PGK1 *mRNA in yeast [35], which allows it to escape NMD. In addition, the APOBEC-1 complementation factor ACF [36], which is present within HepG2 cells, is known to protect edited *APOB *mRNA from NMD [37], and it is possible that this protection is extended to the skip 27 mRNA.

**Figure 6:**
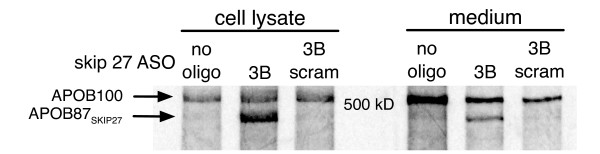
**Transfection of *APOB *skip 27 ASOs causes expression of a short isoform of APOB, APOB87_SKIP27_**. HepG2 cells were transfected with the indicated ASOs at 250 nM, then pulse-labelled with Trans-^35^S-Label for 1 hour in the presence of oleic acid-BSA, which stimulates APOB100 expression [62]. Cell lysates or cell culture media were immunoprecipitated with a polyclonal antibody for APOB. The immunoprecipitates were subjected to SDS-PAGE (4% Tris-glycine), followed by autoradiography. The gel shown is representative of three experiments. A short isoform of APOB, APOB87_SKIP27_, is seen with the skip 27-3B ASO. This short isoform is not seen with the 3B scram ASO, nor with the no-oligo control. This matches the presence and absence of skip 27 mRNA seen with these ASOs.

As the skip 27 mRNA is not subject to NMD, we considered the possibility that APOB87_SKIP27 _might be translated from the skip 27 mRNA. In order to assess this, ASO-treated HepG2 cells were labelled with ^35^S-methionine/cysteine. The cell lysates and media were immunoprecipitated with anti-APOB, subjected to SDS-PAGE and then to autoradiography. Fig. 6 shows that the cells treated with the skip 27-3B ASO expressed two isoforms: a long isoform corresponding to APOB100 and a short isoform consistent with APOB87_SKIP27_. Both isoforms were recovered from the cell lysates and from the culture medium. Neither the mock-transfected cells, nor the cells treated with skip 27-3B scram expressed APOB87_SKIP27_.

Interestingly, the relative ratios of APOB100 and APOB87_SKIP27 _differed between that found in the cell lysate and that secreted into the medium. In the cell lysate, there appears to be a predominance of APOB87_SKIP27 _over APOB100. In the medium, there is a predominance of APOB100 over APOB87_SKIP27_. This may be due to decreased secretion of APOB87_SKIP27 _relative to APOB100, causing an increased cellular retention of APOB87_SKIP27 _relative to APOB100. This is a finding that parallels the decreased secretion rate of the APOB89 truncation in APOB89 heterozygotes [38]. We conclude, therefore, that the skip 27 mRNA is translated into the APOB87_SKIP27 _isoform and that this protein is secreted into the culture medium along with APOB100.

## Discussion

Due to its role in the assembly of the atherogenic lipoprotein particles LDL and Lp(a), *APOB *has become a prime target for therapeutic interventions to reduce LDL and Lp(a) levels, and therefore perhaps to retard atherosclerosis. Approaches that have been used include over-expression of APOBEC-1 to induce RNA editing of *APOB *mRNA and isoform switching to APOB48 [4], RNase H-mediated degradation of *APOB *mRNA using ASOs [5], and RNA interference with siRNA duplexes [6,7]. We demonstrate in this paper that ASOs targeted to the splice-sites of *APOB *exon 27 are effective in inducing alternative splicing of exon 27, and that this skip 27 mRNA is translated to a short isoform of APOB, APOB87_SKIP27_. We are conscious that HepG2 cells, although they are able to synthesize and secrete APOB, do not assemble normal VLDL or LDL [39]. However, the data presented suggests that it is in principle feasible to induce the skipping of the normally constitutive exon 27 in hepatocytes. Should further work demonstrate that it is possible to do this *in vivo *with injected or ingested ASOs, we speculate that this could potentially form the basis of a therapy to reduce APOB100 expression and to reduce circulating LDL and cholesterol levels.

Our work relies on 2'O-methyl RNA oligonucleotides as splice-site ASOs, and joins a growing list of synthetic nucleic acid-based compounds that have been used to modify splicing [40]. ASOs employing a combination of phosphorothioate and 2'O-methyl RNA modifications have been used to modify dystrophin splicing [11,12], ß-globin splicing [14], and *SMN2 *splicing [41]. Other chemistries such as 2'O-methoxyethyl ASO [42], morpholino ASO [43], peptide-nucleic acid (PNA) compounds [44] and chimeric DNA/locked nucleic acid oligonucleotides [45] represent alternatives that might, in future work, be used to modify *APOB *exon 27 splicing. We have also used PNA-peptide chimeras in an approach termed ESSENCE (exon-specific splicing enhancement by small chimeric effectors). These compounds comprise an antisense sequence that targets the compound specifically to a particular exon. The antisense sequence is linked to RS dipeptide repeats which serve to activate splicing and inclusion of the targeted exon. This approach has been successfully used to modify the alternative splicing in the *SMN2 *pre-mRNA [46] and the *Bcl-x *pre-mRNA [47]. The final choice for the chemistry of a therapeutic compound will depend crucially on several factors, including the efficacy of *APOB *exon 27 skipping, the pharmacokinetics and bioavailability, the efficiency of translocation across the cell and nuclear membranes, and the safety of the compound.

APOB87_SKIP27 _is predicted to consist of the N-terminal 3929 residues of APOB100. As there is a frameshift induced by the skipping of exon 27, part of the exon 28 sequence is translated to a divergent 37 amino acid sequence at the C-terminus. We postulate that this isoform, if assembled into VLDL and LDL, will have similar properties to the C-terminally truncated isoforms that have been described and characterized in kindreds with natural mutations in *APOB *causing hypobetalipoproteinemia. One such mutant isoform, APOB89, is produced by a single nucleotide deletion in exon 29, and lowers circulating cholesterol levels to the 5^th ^percentile [48,49]. Patients heterozygous for APOB89 synthesize and secrete APOB89 at 92% of the rate of APOB100, but LDL particles containing APOB89 show greater fractional catabolic rates compared to those containing APOB100, most likely due to the greater affinity of APOB89-containing particles for the LDL receptor [38]. APOB89 also appears to have a dominant negative effect on the secretion rate of APOB100, reducing the production rate in APOB89 heterozygotes to 30% compared to normal individuals [50]. The combination of these phenomena, therefore, leads to a disproportionate decrease in LDL and cholesterol levels in APOB89 heterozygotes. Deletion of the C-terminal 10% of the protein renders APOB unable to assemble into Lp(a) [51]. Consistent with this, in APOB89 heterozygotes, APOB89 is not associated with the Lp(a) particles in these patients. However, Lp(a) levels are not decreased in these individuals. The circulating Lp(a) particles contain APOB100 exclusively, and are indistinguishable from normal Lp(a) in terms of size and density [52].

Another mutant isoform seen in patients with familial hypobetalipoproteinemia, APOB87_Padova_, is caused by a single nucleotide deletion at the 5' end of exon 28, leading to a shortened isoform comprising the N-terminal 3968 residues of *APOB *plus a divergent C-terminal 37 amino acid sequence. This C-terminal sequence, which results from the frameshift of exon 28 sequence introduced by the deletion, is identical over 36/37 residues with the C-terminal divergent sequence of APOB87_SKIP27_, as the same frameshift is introduced by skipping exon 27. Consequently, APOB87_Padova _is an isoform that is very similar to APOB87_SKIP27_, the principal difference being that APOB87_Padova_includes the APOB sequence coded by exon 27. As with APOB89, the APOB87_Padova_-containing LDL from these patients exhibits an increased fractional catabolic rate and decreased production compared to normal individuals, leading to cholesterol levels about 2/3^rd ^normal and LDL levels of about 50% normal in heterozygous individuals [53]. If it were possible to partially induce APOB87_SKIP27 _expression *in vivo*, this could cause a decreased secretion rate of APOB100, and that APOB87_SKIP27_-containing VLDL, IDL and LDL would be cleared at a faster rate than normal, leading to decreased levels of these particles. However, since these ASOs only induce partial exon 27 skipping, APOB100 would still be synthesized and assembled into Lp(a). Therefore, we would also predict that this would not change Lp(a) levels. We can foresee one theoretical problem with our therapeutic approach. Should it be possible to induce APOB87_SKIP27 _*in vivo*, the presence of the 37 amino acid divergent sequence at the C-terminus could induce an immune response to the novel epitope thus generated. It is unknown at this time as to whether this immune response might have any clinical consequences such as antibody interference with APOB87_SKIP27 _function or an autoimmune response.

A therapy based on APOB87_SKIP27 _induction may have particular utility in patients with familial hypercholesterolemia, such as those with familial defective APOB100 (FDB). This condition is due to point mutations that disrupt the conformation of the LDL receptor-binding domain of APOB, leading to decreased affinity for the receptor, decreased clearance of VLDL/IDL/LDL particles, hypercholesterolemia, and therefore an increased risk of atherosclerotic disease [8]. The most common mutation, R3500Q, appears to be quite prevalent in Western and Central European populations, with a heterozygote prevalence of ~1/250 in Switzerland [54]. C-terminal deletions of *APOB *restore the ability of FDB LDL particles to bind to the LDL receptor [55,56]. We predict, therefore, that induction of APOB87_SKIP27 _in patients with FDB would be expected to restore the ability of APOB87_SKIP27_-containing LDL to bind to its cognate receptor. Furthermore, we speculate that patients with familial hypercholesterolemia due to LDL receptor mutations may benefit from the induction of APOB87_SKIP27 _by increasing the affinity of APO87-containing LDL for the LDL receptor.

The various methods that have so far been proposed for down-regulating APOB100 expression have encountered various side-effects, some of them of a serious nature. For example, the over-expression of APOBEC-1 in mouse livers led to liver dysplasia and hepatocellular carcinomas, a phenomenon that has been attributed to promiscuous editing of mRNAs other than *APOB *[4]. Another problem common to those therapeutic interventions that generally down-regulate *APOB *mRNA levels is that they have the potential to repress both APOB100 and APOB48 expression. In the case of RNA interference by cholesterol-conjugated siRNA, APOB48-containing chylomicron levels are reduced by approximately 50% [6]. The ASO ISIS 147764, when administered to mice, down-regulates the *APOB *mRNA and causes a 90% reduction in circulating and liver levels of both APOB100 and APOB48, although, interestingly, no reduction in APOB48 protein levels is seen in the intestine [5]. This reduction in APOB48 expression could interfere with chylomicron assembly and potentially cause fat malabsorption from the intestine, a serious side effect. Induction of exon 27 skipping by ASOs, however, might not be subject to this problem, as the RNA editing site resides within exon 26. The skip 27 mRNA should therefore be edited in the intestine, and translated to result in APOB48. Consistent with this supposition, patients with APOB89 familial hypobetalipoproteinemia produce intestinal APOB48 as normal [57].

## Conclusion

High circulating LDL and cholesterol levels are associated with atherosclerosis, and therapeutic interventions to reduce these levels have been shown to decrease the incidence of the complications of atherosclerosis, such as heart attacks and strokes. Despite modern treatments such as HmG CoA reductase inhibitors, there remains a demand for other interventions to reduce LDL and cholesterol levels. Using ASO-induced alternative splicing of *APOB *mRNA, we have demonstrated that it is possible to induce the expression of a novel isoform of APOB that simulates hypobetalipoproteinemia, and which should reduce circulating LDL and cholesterol levels. This represents a new and promising technique that may therefore have applications in therapeutic modification of APOB100 expression.

## Methods

### Cell culture and transfection of antisense oligonucleotides

2'O-methyl antisense oligonucleotides (ASOs – see Table 1) were ordered from Dharmacon (Lafayette, CO). HepG2 cells were maintained in Minimum Essential Medium with Earle's salts and glutamine, 10% fetal bovine serum (Gibco/Invitrogen, Carlsbad, CA) at 37°C under 5% CO_2_. ASOs were transfected in 12-well plates, with 1 × 10^6 ^cells per well, using 2.4 μl Lipofectamine 2000 in 200 μl OptiMEM-I (Invitrogen) as per the manufacturer's protocol, then incubated for 48 hours before harvesting. A time-course revealed that the ASO effects were seen as early as 24 hours and lasted up to 72 hours. No ASO effects were seen at 96 or 120 hours. The optimal effects were seen at 48 hours. ASOs skip 27-3B and skip 27-3B scram were also labelled with fluorescein at the 5' end and examined after transfection with a Nikon Eclipse microscope with a FITC filter set to control for oligonucleotide transfection efficiency.

### RNA isolation and RT-PCR

Total RNA was isolated from cells using the RNeasy Micro kit (Qiagen, Valencia, CA). The RNA was quantified using the QuantIt Ribogreen kit (Invitrogen), and 2.5 μg of total RNA was reverse-transcribed using Transcriptor reverse transcriptase (Roche Applied Science, Indianapolis, IN) with an oligo dT_15 _primer as per the manufacturer's protocol. 0.25 μl of the cDNA was subjected to PCR with 200 μM of each oligonucleotide and FastStart Taq (Roche Applied Science) using the following program: 95°C for 30 sec, 58°C for 30 sec, 72°C for 30 sec. 0.5 μCi of [α-^32^P] dCTP (Perkin-Elmer, Wellesley, MA) was included per 10 μl reaction. Under these conditions, the number of cycles was adjusted for each oligonucleotide pair to allow amplification to the exponential phase. The sequences of the oligonucleotide pairs are given in Table 2. The PCR reaction was then subjected to PAGE on a non-denaturing 8% gel, dried and autoradiographed. Quantitation was carried out using a Molecular Dynamics Typhoon PhosphorImager and ImageQuant software (GE Healthcare Life Sciences, Piscataway, NJ). The quantified bands were normalized according to the G+C content, and the percentage alternative splicing was calculated as isoform/total of all isoforms. Statistical analysis and graphing was carried out using Prism 4.0c software (GraphPad Software, San Diego, CA).

### Computational identification of exonic splicing enhancers

ESEfinder [21,58], RESCUE-ESE [22,59] and PESXs server [23,24,60] were used to search the *APOB *exon 27 sequence for putative exonic splicing enhancers.

### Calculation of RNA secondary structure

The mFold web server [17,61] was used to calculate the potential secondary structures formed by the *APOB *exon 27 and adjacent intronic sequences.

### Live labeling of HepG2 cells

10 cm plates of HepG2 cells were transfected with the indicated ASOs at 250 nM for 48 hours. After washing the cells with 1× PBS + Complete Protease Inhibitors (Roche Applied Science), the medium was replaced with 4 ml Dulbecco's Modified Eagle's Medium lacking methionine and cysteine (Invitrogen) with 0.5 mM oleic acid-1.6% BSA (Sigma, St Louis, MO). The cells were incubated for 15 min before 200 μCi of Trans-S^35^-Label (MP Biomedicals, Irvine, CA) was added. The cells were incubated for 60 min, the pulse medium was removed, and the cells were washed with 1× PBS + Complete Protease Inhibitors. For the chase, 4 ml of complete DMEM + 1.6% BSA (Invitrogen) was added, and the cells were incubated for 4 hours. Both the pulse and chase media were combined and subjected to concentration using an Amicon Centricon YM-30 (Millipore, Billerica, MA). The cells were scraped off and lysed in 1 ml modified RIPA (50 mM Tris-HCl pH 7.4; 0.5% sodium deoxycholate; 150 mM NaCl; 1 mM EDTA, pH 8.0; 1% Triton X-100; 0.1% SDS, 1 mM PMSF; 1× Complete EDTA-free Protease Inhibitors), then incubated overnight at 4°C on a rotating mixer. After microcentrifugation for 5 mins, the cell lysate supernatant was retained.

The concentrated media were diluted to 12 ml with modified RIPA. 40 μl of polyclonal anti-APOB (AB742: Chemicon International, Temecula, CA) was added to the diluted media and the cell lysate supernatant. After incubation on a rotating mixer for 2 hours at 4°C, 50 μl of a 1:1 slurry of protein A-Sepharose 6 MB in modified RIPA (GE Healthcare Life Sciences) was added and incubation was continued for 5 hours. The beads were washed three times in 1 ml modified RIPA before being boiled with 50 μl 2× SDS-PAGE loading buffer and subjected to SDS-PAGE on a 4% Tris-glycine gel.

## Authors' contributions

BK carried out the experiments, the splice-site and ESE searches, and drafted the manuscript. XR carried out the SpliceRack database search, participated in the design of the study, and helped to draft the manuscript. SLC and ARK revised the manuscript critically for content. All authors read and approved the final manuscript.

## Supplementary Material

Additional file 1 **Two potential secondary structures formed by exon 27 and adjacent intronic regions**. The structures were calculated by mFold [17,61]. Lowercase letters denote intronic sequences, uppercase letters denote exon 27 sequence. The splice-sites are enclosed in grey-shaded boxes (5' splice-site = 5'ss; 3' splice-site = 3'ss; branch-point sequence = BPS). The site of binding of the skip 27-3B ASO is denoted by the black arrow adjacent to the lower limb of both structures. Click here for file

Additional file 2 **Abrogation of NMD with cycloheximide does not increase the levels of *APOB *skip 27 mRNA**. HepG2 cells transfected with the indicated skip 27 ASOs at 250 mM were treated with ethanol vehicle (-) or cycloheximide at 100 μg/ml (+) for 1 hour. No significant difference in skip 27 mRNA levels was observed with cycloheximide treatment. Click here for file

## Figures and Tables

**Table 1:** Sequences of antisense oligonucleotides (ASOs) employed in the experiments

**ASO name**	**Sequence (5'-3') ACGU = 2'O-methyl RNA bases, acgu = RNA bases**
skip 27-5	AUAAAAGCUCCAUACUGAAG
skip 27-3	CAAAACUGUAUAGGAGAGAU
skip 27–53	AUAAAAGCUCCAUACUGAAGCAAAACUGUAUAGGAGAGAU
skip 27-5B	AUAAAAGCUCCAUACUGAAGAUUUUAUUAGAUUCAUAACA
skip 27-3B	CUGUAUAGGAGAGAUUUUGUAUUUUAUUAGAUUCAUAACA
skip 27-3B scram	CAAGUAGAUAUUAUAUGUUGUUACUUGUAUAUAUCGAAUG
skip 27-B	AUUUUAUUAGAUUCAUAACA
skip 27–53 A1	AUAAAAGCUCCAUACUGAAGCAAAACUGUAUAGGAGAGAUucgaguaugauagggacuuagggug
skip 27–53 mut A1	AUAAAAGCUCCAUACUGAAGCAAAACUGUAUAGGAGAGAUucgaguaugauacgcacuuacgcug
skip 27-3B A1	CUGUAUAGGAGAGAUUUUGUAUUUUAUUAGAUUCAUAACAucgaguaugauagggacuuagggug
skip 27-3B mut A1	CUGUAUAGGAGAGAUUUUGUAUUUUAUUAGAUUCAUAACAucgaguaugauacgcacuuacgcug
skip 27-ex 6–25	UCUUCGAUUUUGUGUGUUCC
skip 27-ex 31–50	UUUAGUCUUAGAGGCUAACG
skip 27-ex 61–80	UGCACUGAAGUCACGGUGUG
skip 27-ex 88–107	UUCAUAUUUGCCAUCUUCUU
skip 27-ex 6–25 scram	CUGUUUGUUUCUGACGCUUU
skip 27-ex 31–50 scram	CCAUGGGUCUUUUAGUAAAG
skip 27-ex 61–80 scram	UGGACAUCGCAGACGUUGGU
skip 27-ex 88–107 scram	GUUCUCUUUUUCAUUCCAUA
FLuc A1	GUCUGCUCGAAGCGGCCGGCucgaguaugauagggacuuagggug

**Table 2:** Sequences of oligonucleotides used for RT-PCR

**Exon**	**Cycles**	**Forward**	**Reverse**
*APOB *exon 27	26	CCTTGATTCCCTTTTTTGAGA	GCTCTAGACAGATGGAGATCGGT
GAPDH	22	AAGGTGAAGGTCGGAGTCAACGG	CCACTTGATTTTGGAGGGATCTC
*TSC2 *exon 25	34	GGCCTGCGGTCCAATGTCCTC	CTCTTCGGGACAGCCGTGAAG
Caspase-9 exons 3–6	34	GCTCTTCCTTTGTTCATCTCC	CATCTGGCTCGGGGTTACTGC
Insulin receptor exon 11	32	AACCAGAGTGAGTATGAGGATTCG	TTCTCAAAAGGCCTGTGCTCCTCC
